# Supplementary material for: Multiplexed action-outcome representation by striatal striosome-matrix compartments detected with a mouse cost-benefit foraging task
Source: Nat Commun. 2022 Mar 22;13:1541. doi: 10.1038/s41467-022-28983-5 (PMC8941061; doi:10.1038/s41467-022-28983-5)
Supplement: Supplementary file 1 — Supplementary Information [file 41467_2022_28983_MOESM1_ESM.pdf]

Inventory of Supporting Information

Title: Multiplexed action-outcome representation by striatal striosome-matrix compartments detected with a mouse cost-benefit foraging task

Authors: Bernard Bloem, Rafiq Huda, Ken-ichi Amemori, Alexander Abate, Gaya Krishna, Anna Wilson, Cody W. Carter, Mriganka Sur, Ann M. Graybiel

Supplemental Figures:

Supplementary Fig. 1 Effect of cannula implantation on behavioral performance.
Supplementary Fig. 2 Action-outcome association representations by SPNs.
Supplementary Fig. 3 Activity and selectivity of reward/no-reward/puff/no-puff neurons.
Supplementary Fig. 4 Comparison of cost-benefit reinforcement learning models.
Supplementary Fig. 5 Movement-related activity in sSPNs and mSPNs.
Supplementary Fig. 6 Decoding action and outcome combinations with striatal activity.
Supplementary Fig. 7 Decoding future behavior with striatal activity.

Additional supplemental information:

Source data: source_data.zip
Cost-benefit reinforcement learning model: https://github.com/bloemb/CBC_RL_model

SUPPLEMENTARY FIGURES

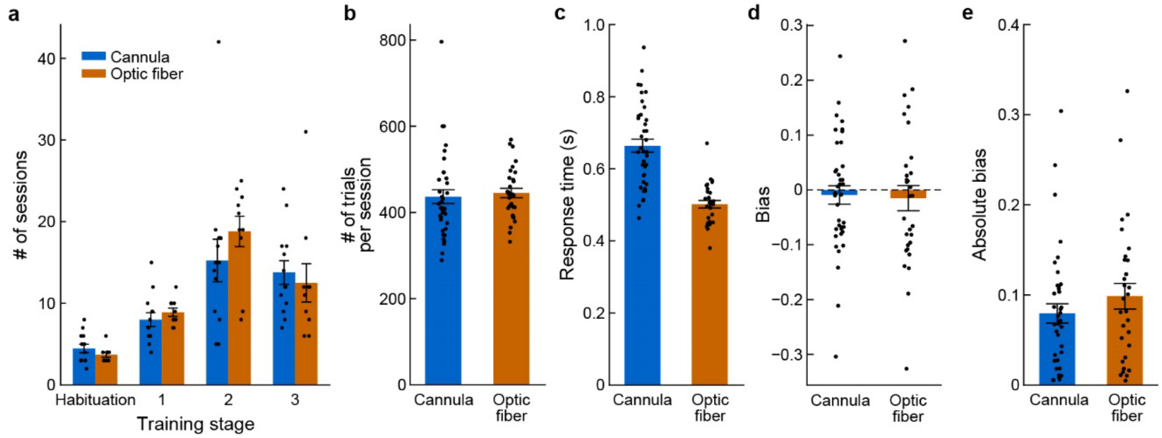

Supplementary Fig. 1 Effect of cannula implantation on behavioral performance. a Number of sessions that was required to progress through all training stages, as described in Methods (mean ± SEM, n = 10 control mice and 12 mice with cannula). **b** Average number of trials performed in the last three sessions before reaching the final performance criterion (mean ± SEM, control: n = 30, cannula: n = 36). **c** Average response time in the last three sessions before reaching criterion (mean ± SEM, control: n = 30, cannula: n = 36). **d** Average bias in the last three sessions before reaching criterion (mean ± SEM, control: n = 30, cannula: n = 36). **e** Average absolute bias in the last three sessions before reaching criterion (mean ± SEM, control: n = 30, cannula: n = 36). Source data are provided as a Source Data file.

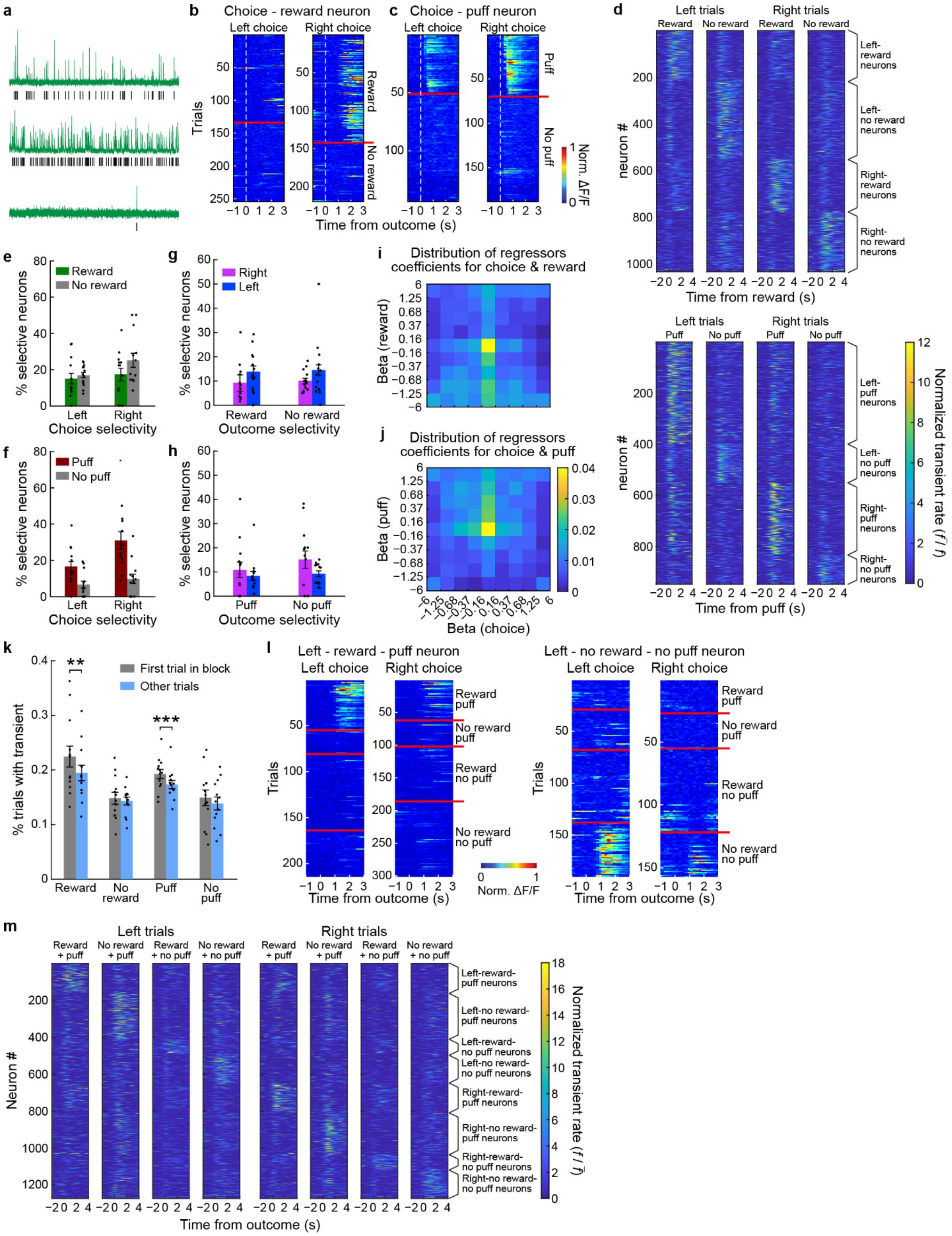
a
b
Choice - reward neuron
Left choice
Right choice
Reward
No reward
Trials
c
Choice - puff neuron
Puff
No puff
Norm. ΔF/F
Time from outcome (s)
d
Left trials
Right trials
Reward
No reward
Left-reward neurons
Left-no reward neurons
Right-reward neurons
Right-no reward neurons
neuron #
Time from reward (s)
Puff
No puff
Left-puff neurons
Left-no puff neurons
Right-puff neurons
Right-no puff neurons
Normalized transient rate
Time from puff (s)
e
% selective neurons
Choice selectivity
f
g
Right
Left
Outcome selectivity
h
i
Distribution of regressors coefficients for choice & reward
Beta (reward)
j
Distribution of regressors coefficients for choice & puff
Beta (puff)
Beta (choice)
k
First trial in block
Other trials
% trials with transient
l
Left - reward - puff neuron
Left - no reward - no puff neuron
Reward puff
No reward puff
Reward no puff
No reward no puff
m
Reward + puff
No reward + puff
Reward + no puff
No reward + no puff
Neuron #
Left-reward-puff neurons
Left-no reward-puff neurons
Left-reward-no puff neurons
Left-no reward-no puff neurons
Right-reward-puff neurons
Right-no reward-puff neurons
Right-reward-no puff neurons
Right-no reward-no puff neurons
Time from outcome (s)

Supplementary Fig. 2 Action-outcome association representations by SPNs. a ΔF/F fluorescent traces of three sample neurons recorded over 30 min (green) and the time of detected Ca^{++} events (black). **b, c** Two examples of neurons showing activity selective for action-reward (**b**) and action-puff (**c**) combinations. Trials are shown (rows) separately for left/right action, with red lines demarcating reward/no-reward or puff/no-puff outcome trials. **d** The analysis for Fig. 2e was repeated, but with half of the trials used for detecting the neuronal response types and the other half for calculating the average response in the different trial types. The results confirm the validity of the detected neuron types. **e** Percentage (mean ± SEM) of action-selective neurons with selectivity for reward or no-reward outcomes (left: reward = 14.9 ± 2.9%, no reward = 16.8 ± 1.6%; right: reward = 17.2 ± 3.4%, no reward = 25.0 ± 3.9%; n = 13 mice). There were no significant main effects or interactions (ANOVA). **f** Percentage of action-selective neurons with selectivity for puff or no-puff trials (left: puff = 16.6 ± 2.7%, no puff = 6.6 ± 1.9%; right: puff = 30.9 ± 4.8%, no puff = 9.7 ± 2.6%; mean ± SEM, n = 13 mice). There were significant main effects of puff outcome (p = 0.000024) and choice (p = 0.012; ANOVA). **g** Percentage of reward-outcome-selective neurons with selectivity for left or right actions. No significant main or interaction effects were detected (mean ± SEM , n = 13 mice). **h** Percentage of puff-outcome-selective neurons that was selective for the two actions. No significant effects were detected (mean ± SEM, n = 13 mice). **I, j** Joint distribution of chosen action and reward (**i**) or puff (**j**) regressor coefficients. Horizontal and vertical bins were chosen to divide the non-zero coefficients equally among the bins. **k** Comparison of action-outcome related responses in action-outcome selective neurons in the first trial after a block switch versus other trials with the same action and outcome (mean ± SEM, n = 13 mice, ***p < 0.001). **l** Two examples of neurons showing activity representing an association between an action and both reward and puff outcomes. **m** The analysis of Fig. 2h repeated with half of the trials used for detecting the neuron types and the other half for calculating the average responses. Source data are provided as a Source Data file.

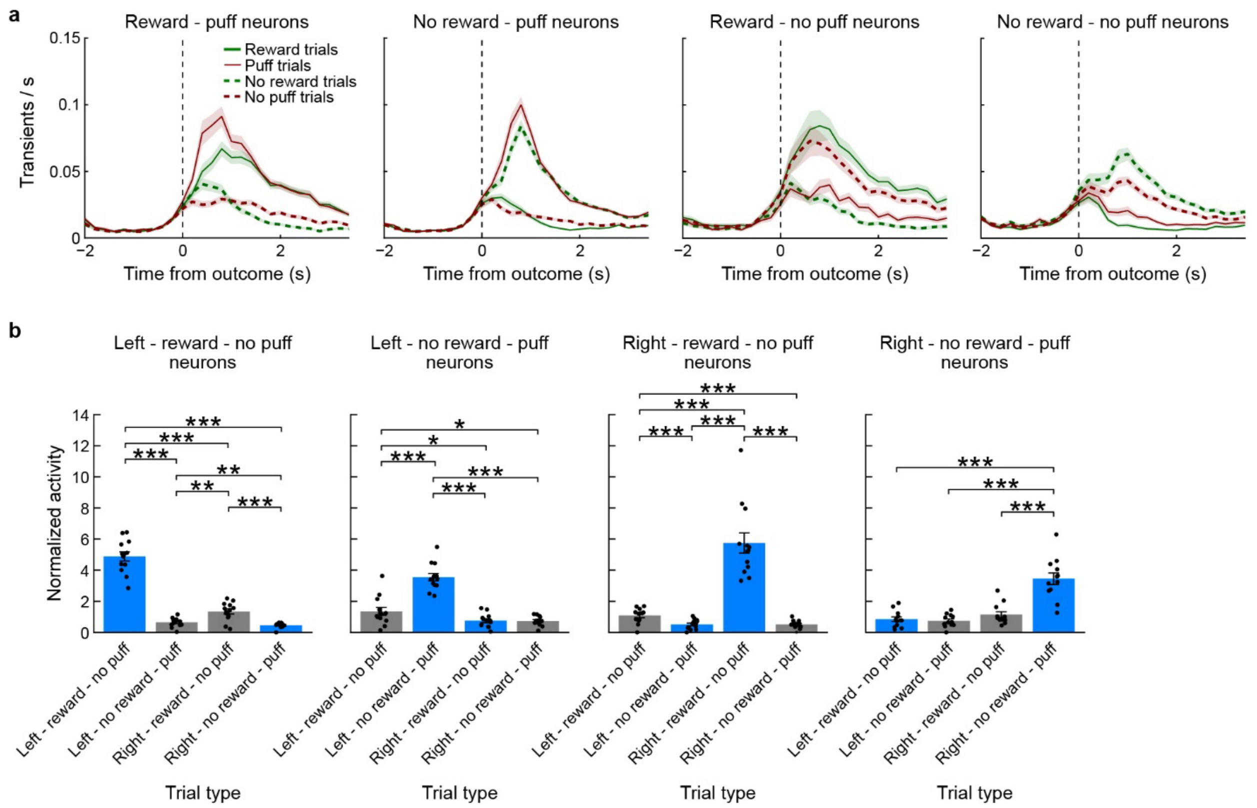

Supplementary Fig. 3 Activity and selectivity of reward/no-reward/puff/no-puff neurons. a Activity (mean ± SEM) of four groups of neurons with activity selective for different outcome combinations in reward/no-reward/puff/no-puff trials (n = 13 mice). **b** Normalized activity of neurons with value-like responses across different trial types (mean ± SEM, n = 13 mice). Neurons that were active in trials in which a good outcome was delivered for one action did not have enhanced activity when the other action was paired with a bad outcome, or vice versa. For all 4 types of neurons, ANOVA indicated significant main effects and interactions ($p < 0.001$). Two-sided post-hoc t-test showed significance between different trial types for each neuron group (Left - no reward - puff neurons: left - no reward - puff trials - left - reward - no puff trials p = 0.00050; Left - no reward - puff neurons: left - no reward - puff trials - right - no reward - puff trials p = 0.025; Left - no reward - puff neurons: left - no reward - puff trials - right - reward - no puff trials p = 0.041; Left - no reward - puff neurons: left - reward - no puff trials - right - no reward - puff trials p = 1e−06; Left - no reward - puff neurons: left - reward - no puff trials - right - reward - no puff trials p = 1e−07; Left - reward - no puff neurons: left - no reward - puff trials - left -

reward - no puff trials p = 1e−09; Left - reward - no puff neurons: left - no reward - puff trials - right - no reward - puff trials p = 1e−07; Left - reward - no puff neurons: left - no reward - puff trials - right - reward - no puff trials p = 1e−09; Left - reward - no puff neurons: left - reward - no puff trials - right - no reward - puff trials p = 0.0064; Left - reward - no puff neurons: left - reward - no puff trials - right - reward - no puff trials p = 0.0078; Left - reward - no puff neurons: right - no reward - puff trials - right - reward - no puff trials p = 0.00036; Right - no reward - puff neurons: left - no reward - puff trials - right - reward - no puff trials p = 0.00023; Right - no reward - puff neurons: left - reward - no puff trials - right - reward - no puff trials p = 0.000055; Right - no reward - puff neurons: right - no reward - puff trials - right - reward - no puff trials p = 0.00067; Right - reward - no puff neurons: left - no reward - puff trials - left - reward - no puff trials p = 0.00075; Right - reward - no puff neurons: left - no reward - puff trials - right - no reward - puff trials p = 0.000018; Right - reward - no puff neurons: left - no reward - puff trials - right - reward - no puff trials p = 0.00045; Right - reward - no puff neurons: left - reward - no puff trials - right - no reward - puff trials p = 1e−05; Right - reward - no puff neurons: right - no reward - puff trials - right - reward - no puff trials p = 1e−05, *p < 0.05; **p < 0.01; ***p < 0.001). Source data are provided as a Source Data file.

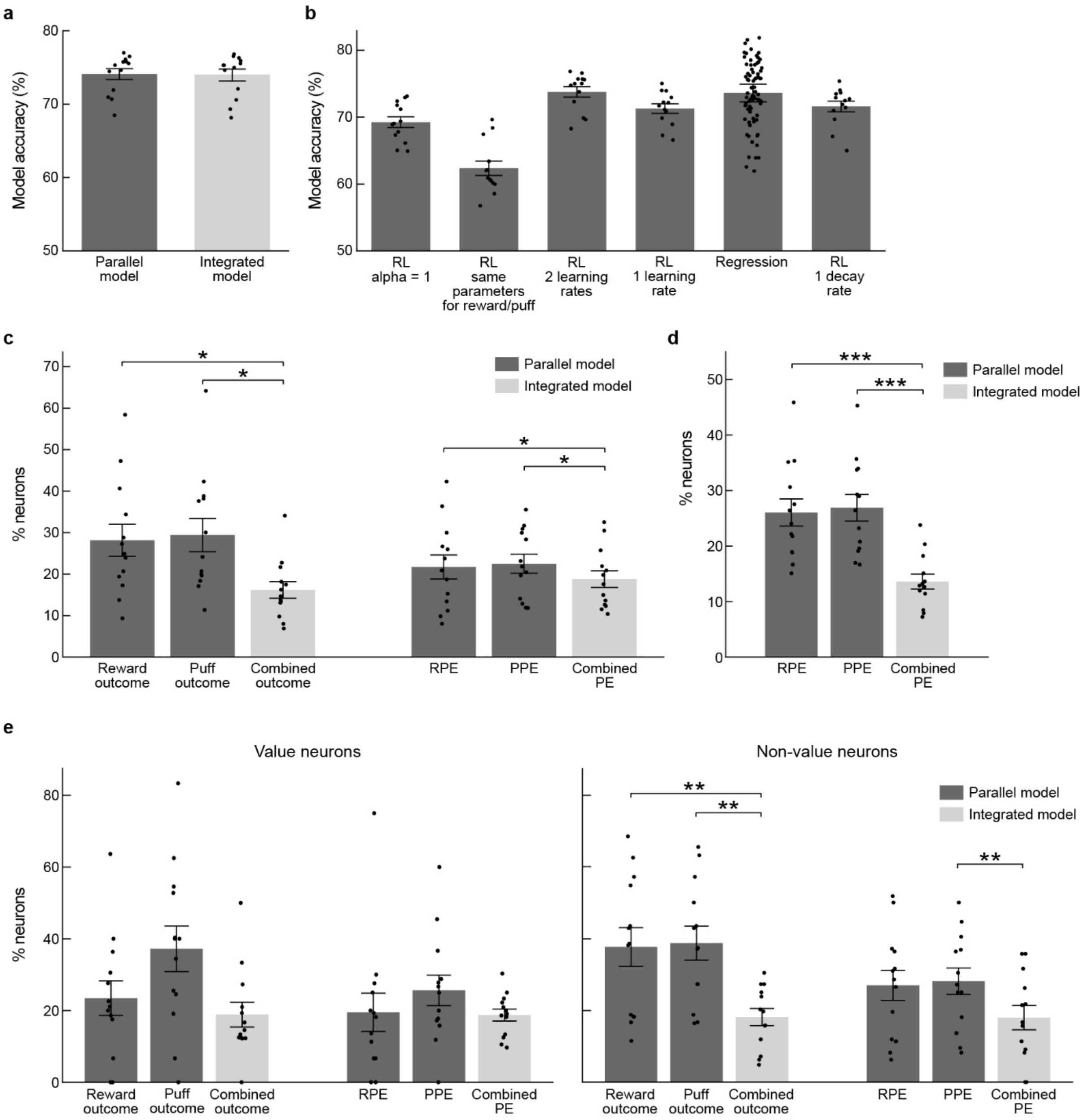

Supplementary Fig. 4 Comparison of cost-benefit reinforcement learning models. a Model accuracy of the parallel and integrated cost-benefit RL models during cross validation (mean ± SEM, n = 13 mice). **b** Cross validation accuracy of alternative simpler models. 'RL alpha = 1': a win-stay/lose-switch model was created by setting all learning rates to 1. 'RL same parameters for reward/puff': one set of learning parameters for both outcomes. 'RL 1 decay rate': there was a single decay rate for both reward and puff. 'RL 2 learning rates: the forgetting rate was set to be the same as the unlearning rate (Ito & Doya, 2009). 'RL 1 learning rate: only 1 learning rate was

used for each outcome. 'Regression': performance of a 5 trial back auto-regressive model. Data shown are mean ± SEM (n = 13 mice for the RL models and n = 75 sessions for the regression model). **c** A stepwise regression was conducted for each neuron to test which factors best account for the recorded activity. The percentage of neurons that include the factors from the two competing cost-benefit RL models was higher for the parallel model (reward outcome vs. combined outcome: $p = 0.011$; puff outcome vs. combined outcome: $p = 0.022$; RPE vs. combined PE: $p = 0.048$; PPE vs. combined PE: $p = 0.035$; $^{*}p < 0.05$, average and SEM of 13 mice; two-sided paired t-test). **d** Partial regression analysis was performed to confirm the results shown in **c** and to quantify the effect of adding prediction errors to models that explain neuronal activity on the basis of outcome. For reward, puff and combined outcomes, we first performed regression of the neurons' activity against, for example, reward. We then regressed RPE against reward, and then finally we regressed the residuals from the first regression against the residuals from the second regression (mean ± SEM, n = 13, $^{***}p < 0.001$). **e** The analysis in **c** was repeated with only neurons that responded to combinations of reward and puff outcomes, and split into 'value' and 'non-value' neurons depending on whether they responded oppositely to reward and puff outcomes or not ($^{**}p < 0.001$). Source data are provided as a Source Data file.

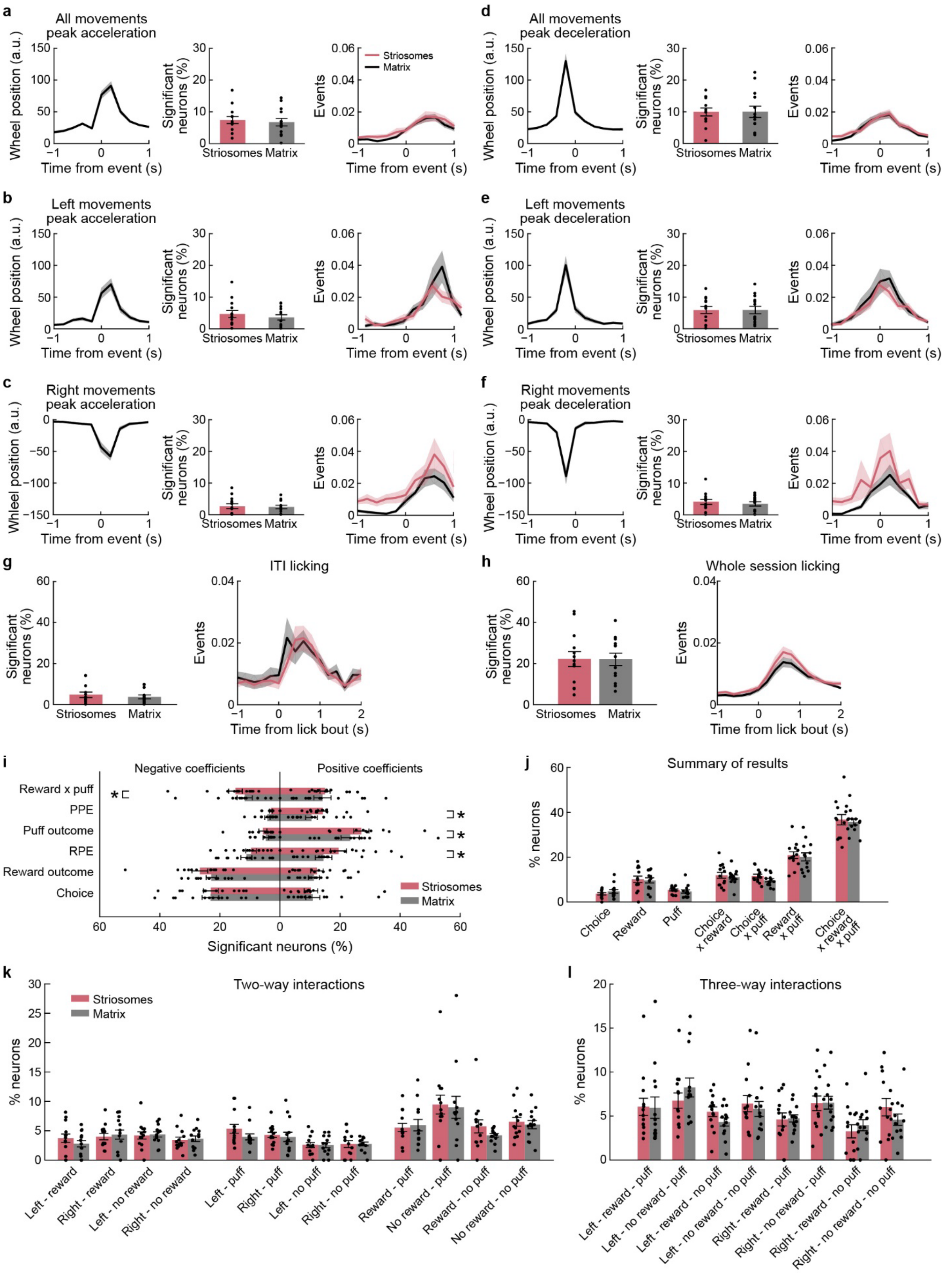
a
All movements peak acceleration
Wheel position (a.u.)
Time from event (s)
Significant neurons (%)
Striosomes
Matrix
Events
b
Left movements peak acceleration
c
Right movements peak acceleration
d
All movements peak deceleration
e
Left movements peak deceleration
f
Right movements peak deceleration
g
ITI licking
Time from lick bout (s)
h
Whole session licking
i
Negative coefficients
Positive coefficients
Reward x puff
PPE
Puff outcome
RPE
Reward outcome
Choice
Significant neurons (%)
j
Summary of results
% neurons
Choice
Reward
Puff
Choice x reward
Choice x puff
Reward x puff
Choice x reward x puff
k
Two-way interactions
Left - reward
Right - reward
Left - no reward
Right - no reward
Left - puff
Right - puff
Left - no puff
Right - no puff
Reward - puff
No reward - puff
Reward - no puff
No reward - no puff
l
Three-way interactions
Left - reward - puff
Left - no reward - puff
Left - reward - no puff
Left - no reward - no puff
Right - reward - puff
Right - no reward - puff
Right - reward - no puff
Right - no reward - no puff

Supplementary Fig. 5 Movement-related activity in sSPNs and mSPNs. a sSPN (red) and mSPN (black/gray) activity was aligned to peak acceleration of the absolute value of wheel movement bouts. Panels show average (± SEM) movements across mice (left), mean proportion (± SEM) of neurons with significant increase in movement-related activity (middle), and mean (± SEM) activity of significantly modulated neurons (right: sSPNs = 7.4 ± 1.1%, mSPNs = 6.7 ± 1.2%; n = 13 mice, two-sided unpaired t-test, p = 0.67, t = −0.43, df = 24). **b, c** Similar to **a**, except movements were divided into left (**b,** sSPNs = 4.6 ± 1.1%, mSPNs = 3.6 ± 0.8%; n = 13 mice, two-sided unpaired t-test, p = 0.47, t = −0.73, df = 24) and right (**c,** sSPNs = 2.7 ± 0.8%, mSPNs = 2.5 ± 0.6%; n = 13 mice, two-sided unpaired t-test, p = 0.81, t = −0.24, df = 24). Data are shown as mean ± SEM. **d** Similar to **a**, except activity was aligned to peak deceleration within wheel movement bouts (sSPNs: 9.9 ± 1.3%, mSPNs: 10.0 ± 1.8%; n = 13 mice, two-sided unpaired t-test, p = 0.99, t = 0.02, df = 24). Data are shown as mean ± SEM. **e, f** Similar to **b** and **c**, except with activity aligned to peak decelaration for left (**e**, sSPNs = 5.9 ± 1.1%, mSPNS = 5.9 ± 1.2%; n = 13 mice, two-sided unpaired t-test, p = 0.99, t = 0.01, df = 24) and right (**f**, sSPNs = 4.1 ± 0.8%, mSPNs = 3.4 ± 0.7%; n = 13 mice, two-sided unpaired t-test, p = 0.53, t = −0.63, df = 24) movements. Data are shown as mean ± SEM. **g** Neuronal activity (mean ±SEM) aligned to licking bout onset during ITI (sSPNs: 4.7 ± 0.9%, mSPNs: 3.7 ± 1.3; n = 13 mice, two-sided unpaired t-test, p = 0.53, t = −0.63, df = 24). **h** Same as **g**, except for licking bouts occurring during the whole session (sSPNs: 22.1 ± 3.6%, mSPNs: 22.0 ± 3.0%; n = 13 mice, two-sided unpaired t-test, p = 0.98, t = −0.02, df = 24). Data are shown as mean ± SEM. **i** Percentage (mean ± SEM) of sSPNs (red) and mSPNs (black/gray) per mouse that included the chosen action, reward and puff outcomes, their interaction, and RPE and PPE in the optimal model using stepwise regression. Significantly more sSPNs included RPE (p = 0.011, t = 2.98, df = 12), puff outcome (p = 0.049, t = 2.19, df = 12), PPE (p = 0.039, t = 2.32, df = 12) and reward x interaction (p = 0.030, t = 2.45, df = 12) in their optimal model (two-sided repeated measures t-test, n = 13, *p < 0.05, n = 13 mice). **j** Summary of stepwise regression showing average percentage of sSPNs and mSPNs per mouse with single action and outcome factors included in their optimal model, as well as different two-way and three-way interactions (mean ± SEM, n = 13). **k, l** Percentage of sSPNs and mSPNs with various two-way (**k**) and three-way (**l**) interactions included in their optimal regression model. There are no significant

differences in any of the comparisons (mean ± SEM, $p > 0.05$, $n = 13$ mice, two-sided repeated measures t-test). Source data are provided as a Source Data file.

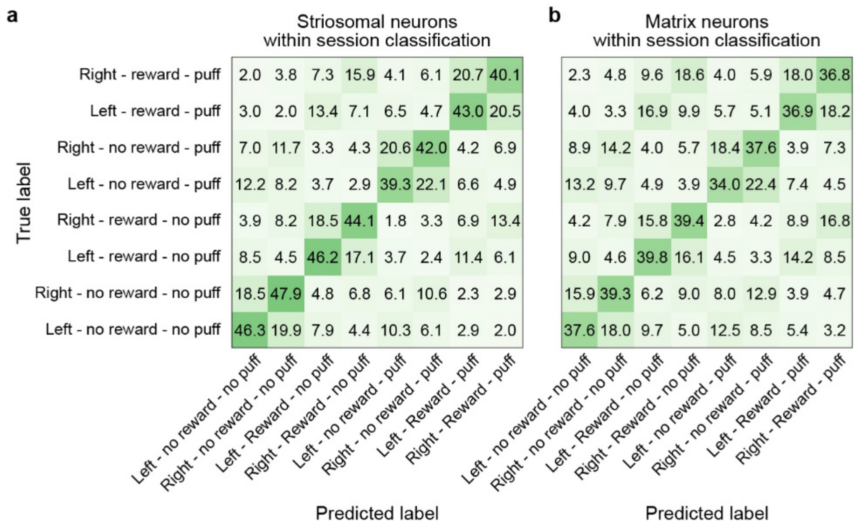

Supplementary Fig. 6 Decoding action and outcome combinations with striatal activity. Confusion matrices for striosomal (**a**) and matrix (**b**) decoding of action – reward outcome – puff outcome combinations using single session models.

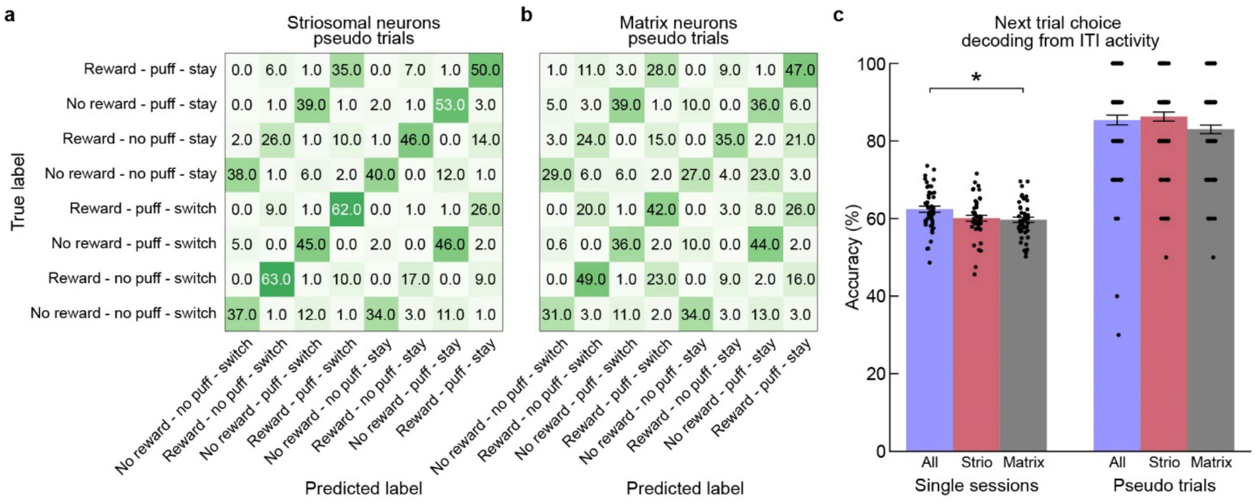

Supplementary Fig. 7 Decoding future behavior with striatal activity. a, b Decoding of future switch/stay behavior and reward and puff outcome in striosomes (**a**) and matrix (**b**) using pseudo trials. **c** Accuracy of decoding left/right choices based on ITI activity in the 2 s preceding trial onset (*p < 0.05). Decoding accuracy in models based on single sessions was slightly better in a model using all neurons than only matrix neurons (mean ± SEM, p = 0.033; t = 2.00, two-sided repeated measures t-test, df = 12, n = 13, 100 pseudo trials). Source data are provided as a Source Data file.
