# Supplementary material for: Multiplexed action-outcome representation by striatal striosome-matrix compartments detected with a mouse cost-benefit foraging task
Source: Nat Commun. 2022 Mar 22;13:1541. doi: 10.1038/s41467-022-28983-5 (PMC8941061; doi:10.1038/s41467-022-28983-5)
Supplement: Supplementary file 2 — Reporting Summary [file 41467_2022_28983_MOESM2_ESM.pdf]

nature portfolio

Corresponding author(s): Ann M. Graybiel

Last updated by author(s): Aug 26, 2021

Reporting Summary

Nature Portfolio wishes to improve the reproducibility of the work that we publish. This form provides structure for consistency and transparency in reporting. For further information on Nature Portfolio policies, see our Editorial Policies and the Editorial Policy Checklist.

Statistics

For all statistical analyses, confirm that the following items are present in the figure legend, table legend, main text, or Methods section.

n/a	Confirmed	
☐	☒	The exact sample size (*n*) for each experimental group/condition, given as a discrete number and unit of measurement
☐	☒	A statement on whether measurements were taken from distinct samples or whether the same sample was measured repeatedly
☐	☒	The statistical test(s) used AND whether they are one- or two-sided<br>*Only common tests should be described solely by name; describe more complex techniques in the Methods section.*
☐	☒	A description of all covariates tested
☐	☒	A description of any assumptions or corrections, such as tests of normality and adjustment for multiple comparisons
☐	☒	A full description of the statistical parameters including central tendency (e.g. means) or other basic estimates (e.g. regression coefficient) AND variation (e.g. standard deviation) or associated estimates of uncertainty (e.g. confidence intervals)
☐	☒	For null hypothesis testing, the test statistic (e.g. *F*, *t*, *r*) with confidence intervals, effect sizes, degrees of freedom and *P* value noted<br>*Give P values as exact values whenever suitable.*
☒	☐	For Bayesian analysis, information on the choice of priors and Markov chain Monte Carlo settings
☐	☒	For hierarchical and complex designs, identification of the appropriate level for tests and full reporting of outcomes
☐	☒	Estimates of effect sizes (e.g. Cohen's *d*, Pearson's *r*), indicating how they were calculated

Our web collection on statistics for biologists contains articles on many of the points above.

Software and code

Policy information about availability of computer code

Data collection	Data was collected using a Prairie Ultima IV 2-photon microscopy system. Behavioral data was collected using MATLAB.
Data analysis	We used MATLAB (2016,2017,2018), Python 3 and Image-J for data analysis. All code will be available upon request

For manuscripts utilizing custom algorithms or software that are central to the research but not yet described in published literature, software must be made available to editors and reviewers. We strongly encourage code deposition in a community repository (e.g. GitHub). See the Nature Portfolio guidelines for submitting code & software for further information.

Data

Policy information about availability of data

All manuscripts must include a data availability statement. This statement should provide the following information, where applicable:

- Accession codes, unique identifiers, or web links for publicly available datasets
- A description of any restrictions on data availability
- For clinical datasets or third party data, please ensure that the statement adheres to our policy

All data will be available upon request

Field-specific reporting

Please select the one below that is the best fit for your research. If you are not sure, read the appropriate sections before making your selection.

☒ Life sciences ☐ Behavioural & social sciences ☐ Ecological, evolutionary & environmental sciences

For a reference copy of the document with all sections, see nature.com/documents/nr-reporting-summary-flat.pdf

Life sciences study design

All studies must disclose on these points even when the disclosure is negative.

Sample size	We used 13 mice, from which we recorded 5831 neurons in 75 experimental sessions.<br>We did not perform a formal power analysis because we did not know how many mice would learn the new behavioral task and also have a good quality imaging preparation.<br>We wanted to analyze the neuronal activity at the level of the mice, not neurons, as the activity of separate neurons in one mouse are not fully independent.<br>Our sample size of n = 13 mice is consistent with previous similar studies.
Data exclusions	Of the mice whose neuronal activity we imaged we did not exclude any mice.<br>We did not include mice that did not learn the task or that did not have clear and stable imaging preparations.
Replication	We performed statistical testing to ensure that the observed effects are consistent across mice.
Randomization	There was only one experimental group.<br>The action-outcome contingencies were randomly set at the beginning of the sessions, and changed after 6-15 rewards or avoided puffs.
Blinding	There was only one experimental group in this study.

Reporting for specific materials, systems and methods

We require information from authors about some types of materials, experimental systems and methods used in many studies. Here, indicate whether each material, system or method listed is relevant to your study. If you are not sure if a list item applies to your research, read the appropriate section before selecting a response.

Materials & experimental systems

n/a	Involved in the study	
☒	☐	Antibodies
☒	☐	Eukaryotic cell lines
☒	☐	Palaeontology and archaeology
☐	☒	Animals and other organisms
☒	☐	Human research participants
☒	☐	Clinical data
☒	☐	Dual use research of concern

Methods

n/a	Involved in the study	
☒	☐	ChIP-seq
☒	☐	Flow cytometry
☒	☐	MRI-based neuroimaging

Animals and other organisms

Policy information about studies involving animals; ARRIVE guidelines recommended for reporting animal research

Laboratory animals	We used 13 Mash1-CreER (het; Ascl1tm1.1(Cre/ERT2)Jejo/J, Jackson Laboratory) x Ai14 (het, B6;129S6-Gt(ROSA)26Sor, Jackson Laboratory). Of the 13 mice, there were 9 female and 4 males adult mice.
Wild animals	Study did not involve wild animals
Field-collected samples	Study did not involve field-collected samples
Ethics oversight	All experiments were conducted in accordance with the National Institute of Health guidelines and with the approval of the Committee on Animal Care at the Massachusetts Institute of Technology

Note that full information on the approval of the study protocol must also be provided in the manuscript.
